# In vivo comparison of initial caries lesions using the enamel decalcification index and quantitative light-induced fluorescence measurement during orthodontic therapy

**DOI:** 10.1007/s00784-025-06234-3

**Published:** 2025-03-10

**Authors:** Priscila Ferrari-Peron, Lisa M. Steuer, Irene Schmidtmann, Ambili R. Mundethu, David Canzler, Heinrich Wehrbein, Christina Erbe

**Affiliations:** 1 Department of Dentofacial Orthopedics & Orthodontics, University Medical Center of the Johannes Gutenberg-University, Mainz, Germany; 2 Institute for Medical Biostatistics, Epidemiology and Informatics (IMBEI), University Medical Center of the Johannes Gutenberg-University, Mainz, Germany

**Keywords:** Initial caries lesions, Orthodontics, Enamel decalcification index, Quantitative light fluorescence, Multibracket appliance

## Abstract

**Objectives:**

To compare two quantitative assessment methods - visual-tactile examination and fluorescence measurement - for detecting of initial caries lesions in adolescents undergoing treatment with a multibracket appliance (MB).

**Materials and methods:**

This study included 28 subjects (14 males, 14 females), treated with MB in both the maxilla and mandible. Data collection occurred at three times points: prior to treatment (T0), six months after MB insertion (T1), and one year post-insertion (T2). The Enamel Decalcification Index (EDI; 0–3 scale) and quantitative light-induced fluorescence (QLF) were employed for assessment.

**Results:**

At T0, four subjects (14%) exhibited no lesions, while only two (7%) remained lesion-free at T1, and again at T2. The kappa coefficient for agreement between the two diagnostic methods across all time points was 0.71.

**Conclusions:**

Both the QLF and EDI methods yielded similar results, with only minor discrepancies. To determine the most appropriate method for each individual case, considerations of cost, benefit and time should be made.

**Clinical relevance:**

The similarity in outcomes for the QLF and EDI methods indicates that both diagnostic methods are effective and reliable. However, QLF may be prone to interference, which must be accounted for during its application.

## Introduction

The primary objective of orthodontic treatment is the early identification and prevention of malocclusion, alongside the optimization of functional occlusion and aesthetic outcomes. Treatment with multibracket appliances (MB) enables efficient tooth movement and shaping of dental arches, while requiring less patient compliance than removable appliances. However, an undesirable side effect of orthodontic therapy, and especially of fixed appliances, is the occurrence of initial caries lesions [1, 2].

Initial caries lesions are areas of enamel demineralization that often manifest as opaque, whitish spots during MB treatment [3, 4]. The main factor contributing to initial caries lesion development is increased plaque accumulation, exacerbated by the presence of brackets, arch wires, and bands, which impede effective oral hygiene [4, 5]. Initial enamel lesions may emerge within four weeks of treatment initiation if oral hygiene is inadequate [4–6], with initial caries lesion progression occuring more rapidly than conventional caries lesions [5], which typically develop over months [7]. The prevalence of initial caries lesions during orthodontic treatment with fixed appliances has been reported to range between 2% and 96% [1, 8–13]. Beyond aesthetic concerns, initial caries lesions increase the risk of caries, particularly post-debonding, necessitating potential restorative treatment [14–16].

Early detection of initial lesions is critical for ensuring the success of orthodontic treatment, preserving both dentofacial aesthetics and structural integrity post-orthodontics. Conventionally used visual-tactile examination methods, such as the International Caries Detection and Assessment System (ICDAS) [17], have been widely utilized and extensively studied. Initially developed in 2001 by an international group of researchers, ICDAS was introduced as a standardized framework to integrate modern caries detection methodologies into a unified system. The system draws upon concepts from prior research by Ekstrand et al. [18, 19], Fyffe et al. [20], and other caries detection methods highlighted in a systematic review conducted by Ismail [21].

ICDAS classifies caries lesions based on their clinical visual appearance, assigning scores ranging from 0 to 6 ranging from a sound tooth surface to an extensive cavity with visible dentin. Despite its strengths, ICDAS does not provide information about lesion localization. Consequently, the Enamel Decalcification Index (EDI) was employed in this study as an alternative to address this limitation.

Visual-tactile examination methods, such as ICDAS and EDI, are now being complemented by promising techniques like quantitative light-induced fluorescence (QLF). While the visual detection of chalky, whitish opacities typically occurs at later stages of demineralization, QLF enables earlier detection, offering clinicians the opportunity for timely and individualized interventions [22, 23].

The aim of this prospective clinical study is to quantitatively assess the occurrence of initial caries lesions in adolescents during the first year of MB initiation using the EDI and QLF. The study also aims to evaluate the correlation of these two diagnostic methods.

## Materials and methods

The protocol was reviewed and approved by the ethics committee of the State Medical Board of Rhineland-Palatinate, Mainz, Germany (Ref: 2020–14928). All procedures were conducted in compliance with the International Conference on Harmonization’s Good Clinical Practice guidelines and the European Union’s Commission Directive 2005/28/EC. Written informed consent was obtained from all participants and their legal guardians.

### Subjects and preparation of the study

The study enrolled 28 subjects (14 female, 14 male) undergoing treatment for tooth position and/or bite anomalies with MB appliances in both the maxilla and mandible.

All treatments were carried out by a trained and experienced examiner (L.S.) at the Department of Dentofacial Orthopedics and Orthodontics, University Medical Center of the Johannes Gutenberg-University, Mainz, Germany. The mean age of the subjects was 13.9 ± 1.3 years, with 96% (*n* = 27) brushing their teeth using their right hand. Data were collected at three distinct time points over an average observation period of 370 ± 34.5 days.

T0 = Baseline, prior to MB appliance placement.

T1 = six months after MB appliance placement.

T2 = twelve months after MB appliance placement.

The buccal surfaces of the four anterior teeth, canines and premolars were examined. Surfaces that could not adequately assessable by both diagnostic methods (QLF and EDI) were excluded, leaving 519 tooth surfaces at T0, 432 at T1, and 423 at T2, totaling 1.374 surfaces for analysis. A surface was classified as affected, if a lesion was detectable by either diagnostic method.

**Fig. 1:**
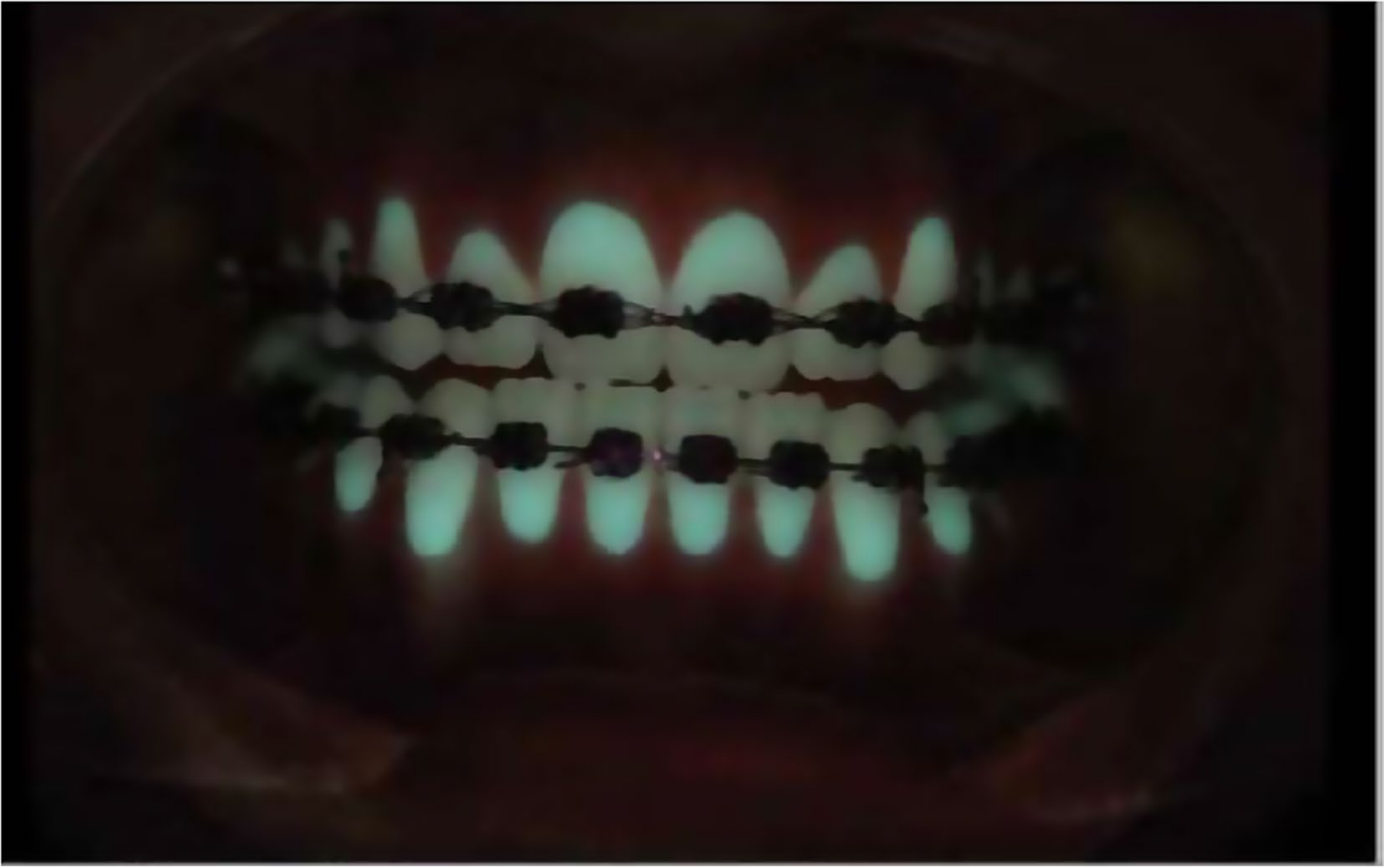
Clinical QLF Image

Prior to data collection, mechanical supragingival plaque and dental calculus were removed using oscillating sonication and polishing with fluoride-free paste (Zircate^®^ Prophy Paste, Dentsply De Trey GmbH, Constance, Germany). This was followed by a visual assessment with a mirror and dental light source, and determination of the EDI. Subsequently, QLF images (Fig. 1) were acquired. To optimize image quality and prevent interference from saliva, tooth surfaces were air-dried for five seconds before each diagnostic procedure. To assess the reproducibility of the diagnostic methods, data from five randomly selected participants were reanalyzed four to six weeks after the first analysis. QLF images were reevaluated, and digital images were used to reassess EDI values.

### EDI according to banks

The bracket-free surfaces of the teeth were divided into four areas – gingival (g), occlusal (o), mesial (m) and distal (d) and visually assessed according to the severity of decalcification:

Grade 0 = No visible decalcification.

Grade 1 = Clinically visible decalcification < 50% of the areas under consideration.

Grade 2 = Moderate to serious decalcification > 50% of the area under consideration.

Grade 3 = Decalcification occupying the entire area under consideration or surface cavities due to advanced caries.

Surfaces obscured by gingival hyperplasia or residual bonding material were excluded from the analysis as “unassessable”.

### QLF measurement

An SLR camera (Canon EOS 550D), equipped with a 60 mm macro lens and a mounted biluminator featuring concentric white and blue light-emitting diodes (QLF-D Biluminator^TM^2, Inspector Research Systems, Amsterdam, Netherlands) was employed for the detection of initial caries lesions. Images were captured in a darkened environment, maintaining a 20 cm distance between the camera lens and the tooth surface. For each clinical visit, three images were obtained from frontal, right-lateral and left-lateral perspectives, with the teeth in edge-to-edge position. The QLF technique utilizes blue light, peaking at 405 nm, to induce bioluminescence in dental tissue, thereby facilitating the detections of demineralized areas. In initial caries lesions, the crystalline structure of the enamel is compromised, which contrasts with intact enamel. As a result, the fluorescent carriers excited by the blue light emit light that is absorbed as it passes through the structurally altered lesion, rendering it visible as a dark region in the QLF image. Lesion dimensions were quantified using QA2 software version 1.26 (Inspector Research Systems, Amsterdam, The Netherlands).

Each pixel in the selected area was assigned a new value based on a regenerated reference standard. Lesions exhibiting fluorescence loss appeared in varying shades of grey, with darker shades indicating greater fluorescence loss and, consequently, more advanced demineralization. Table 1 presents the analytical initial caries lesion values derived from this study. Fluorescence loss relative to lesion volume was calculated using the formula $\:\varDelta\:F=\varDelta\:QxWSArea$.

**Table 1:** Definitions of the QLF-derived values

Measurement	Unit	Description
**ΔF**	%	Fluorescence loss, relative to lesion depth
**ΔF max**	%	Maximum fluorescence loss in measured lesions (highest recorded value)
**ΔQ**	%/px^2^	Fluorescence loss, relative to lesion volume
**WS Area**	px^2^	Area of the lesion exhibiting ΔF ≤ 5% threshold

### Sample size consideration

The number of patients was chosen for practical reasons. With 28 patients there would ideally 560 teeth be included in the study. Agreement between the methods on presence or absence of lesions was to be quantified by Cohen’s kappa. A sample size of *n* = 489 would lead to 95% confidence intervals for kappa with limits at most +/- 0.1 from the point estimate under the following assumptions: kappa ≥ 0.4 and prevalence of lesions per tooth of 20% [24]. This allows for a proportion of 13% of teeth not available for evaluation when including 28 patients.

### Statistical evaluation

Statistical analyses were performed using SAS software (Version 9.4 for Windows, SAS Institute, Cary, North Carolina, USA). To assess initial caries lesion prevalence and incidence, positive initial caries lesion findings were categorized according to the diagnostic criteria and recoded in binary form.

The temporal trend in initial caries lesion development, was measured by the EDI over a one-year observation period. EDI per tooth was obtained as mean of the available values of the four surfaces.

Multiple lesions identified on a single surface via QLF were independently analyzed and quantified. To summarize QLF values for a given tooth, the average percent fluorescence loss across all lesions was calculated. To assess the concordance between the two diagnostic methods in detecting initial caries lesions, results of both methods were dichotomized with 0 representing “no lesion”. Subsequently, Cohen’s kappa coefficient was determined for each examination period (T0, T1 and T2) separately and for all three time points combined. Spearman correlation was computed for all observations, i.e. each evaluable tooth at each visit, between mean EDI and QLF measures. Intra-rater reliability for QLF results was assessed using the intraclass correlation coefficient, calculated through a variance component model. For the EDI method, Cohen’s kappa test was applied to assess agreement between the initial in situ visual examination and subsequent evaluation based on digital photographs.

## Results

At baseline (T0), 14% of participants (*n* = 4) were lesion-free, i.e. neither method detected any lesion for these participants, while 86% (*n* = 24) exhibited at least one initial caries lesion prior to the commencement of MB therapy. Six months after the initiation of MB (T1), 7% (*n* = 2) remained lesion-free, whereas 93% (*n* = 25) had at least one detectable lesion. At T2, although the number of lesion-free subjects remained constant at two, a slight shift in percentages was observed due to the inclusion of only 26 subjects in the analysis. One year after the start of MB, 8% of the cohort (*n* = 2) were free from initial caries lesions. Over the course of one year, the number of subjects with one to two lesions diminished, while the proportion of individuals with more than five lesions rose from 47 to 61% over the observation period. The incidence of initial caries lesions was assessed at two points: first, by comparing T0 with T1, and second, by comparing T0 with T2. Lesions were distinguished based on the detection method– either by QLF or visual inspection through the EDI. The percentage of incisional lesions detected by QLF was 12% at T1 (*n* = 60) and increased by an additional 6% at T2 (*n* = 91).

In comparison, the EDI method recorded an incidence of 6% (*n* = 32) at T1, which increased to 10% (*n* = 54) at T2.

**Fig. 2:**
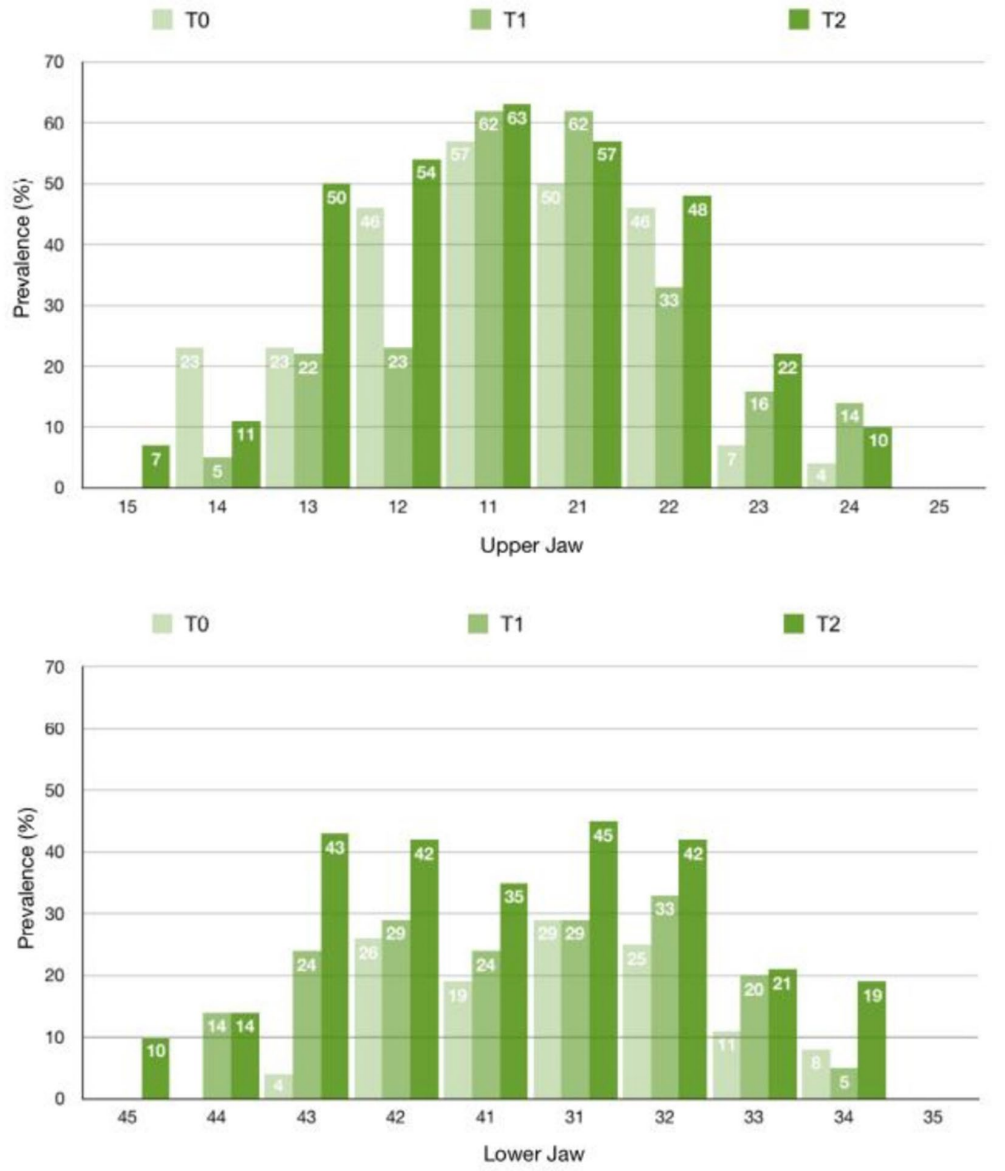
Prevalence of initial caries lesions by tooth and appointment as classified by QLF. The bar charts depict the examined teeth of the maxilla and mandible, numbered according to the scheme of the World Dental Federation FDI, across the examination appointments (T0-T2) along the x-axis, while the y-axis represents the prevalence of initial caries lesions as a percentage

**Fig. 3:**
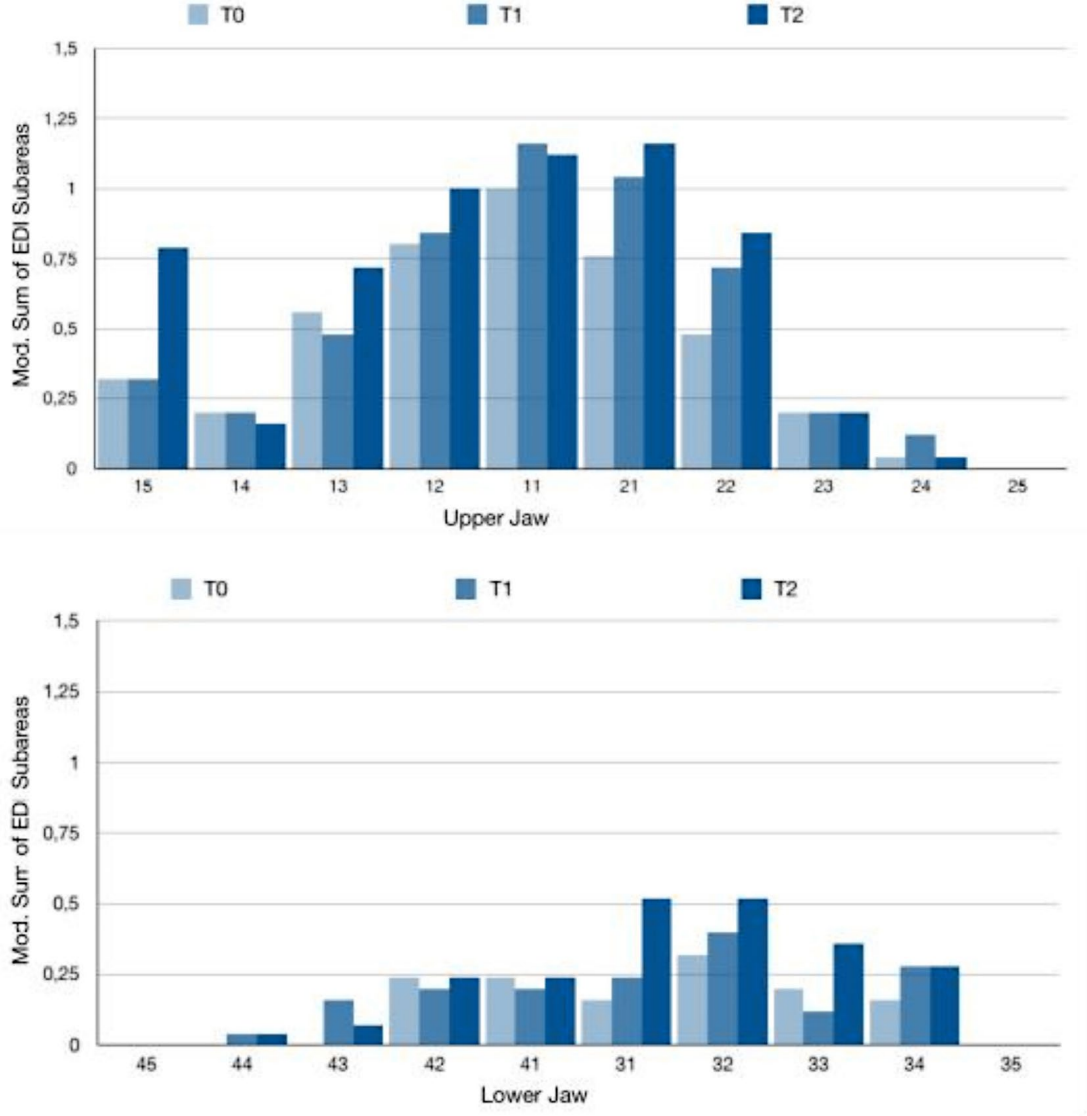
Distribution of initial caries lesions based on EDI assessment per individual tooth. The bar charts display the examined teeth of the maxilla and mandible across the examination appointments (T0-T2) along the x-axis. The y-axis illustrates the mean EDI per tooth, derived from the EDI grades 0–3 for each individual partial tooth surface

The findings from the EDI and QLF diagnostics revealed that the upper incisor region exhibited the highest prevalence and incidence rates, whereas the premolars were consistently the least affected across all examination time points (Figs. 2 and 3). To facilitate the comparison between the two diagnostic procedures, the EDI data and QLF values were recoded into binary variables, and cross-tabulations were performed for each time point.

Baseline data at T0 revealed that 519 buccal tooth surfaces were examined. Of these, no lesions were detected on 402 (77.5%) by either the QLF method or the visual assessment based to the EDI. Six months after MB insertion (T1), both methods found no lesions on 303 surfaces (70.1%), and this number further decreased to 265 tooth surfaces (62.7%). Conversely, positive initial caries lesion findings were identified on 91 surfaces (17.5%) at T0 by both methods. After six months (T1), this number declined to 72 surfaces (16.7%), only to rise again to 96 teeth (22.7%) one year into the treatment. Table 2 summarizes the percentage distribution of the examined tooth surfaces across all three time points, categorized by initial caries lesion findings.

**Table 2:** Comparison of the examination results of both diagnostic methods; percentage of each examined tooth surface type, summarized over all three assessment types, depending on the initial caries lesion type

Lesions detected by QLF	Lesion detected by EDI		
*n* (%)	No	Yes	Total
**No**	970 (70.60)	66 (4.80)	1036 (75.40)
**Yes**	79 (5.75)	259 (18.85)	338 (24.60)
**Total**	1049 (76.35)	325 (23.65)	1374 (100.00)

A positive QLF result coupled with a negative EDI result was documented for 79 of the 1374 observations (i.e. tooth surfaces at each visit) examined. In contrast, both diagnostic methods yielded positive findings in 259 observations.

Tables 3 and 4 again shows the agreement of both methods on the absence of lesions in 970 of 1374 observations. When QLF was below − 8, then mean EDI was at least 1. Conversely, while for mean EDI between 0.25 and 0.75 there were still more than 20% of cases with ΔF = 0, whereas for mean EDI = 1 there was only 1 observation (6.3%) and none for EDI > 1.

**Table 3:** Comparison of categories of ΔF with mean EDI

Table of ΔF by Mean EDI								
ΔF	**EDI**							
n(%)	**0**	**0.25**	**0.5**	**0.75**	**1**	**1.25**	**1.5**	**Total**
< -8	4(0.29)	21(1.53)	4(0.29)	6(0.44)	1(0.07)	2(0.15)	1(0.07)	39(2.84)
(-8, -7]	14(1.02)	35(2.55)	11(0.80)	4(0.29)	3(0.22)	0(0.00)	0(0.00)	67(4.88)
(-7, -6]	42(3.06)	70(5.09)	17(1.24)	6(0.44)	7(0.51)	0(0.00)	0(0.00)	142(10.33)
(-6, -5]	19(1.38)	44(3.20)	17(1.24)	6(0.44)	4(0.29)	0(0.00)	0(0.00)	90(6.55)
0	970(70.60)	46(3.35)	13(0.95)	6(0.44)	1(0.07)	0(0.00)	0(0.00)	1036(75.40)
Total	1049(76.35)	216(15.72)	62(4.51)	28(2.04)	16(1.16)	2(0.15)	1(0.07)	1374(100.00)

**Table 4:** Correlation of EDI and QLF measures

		ΔF	ΔFmax	ΔQ	WS area
All observations	*n* = 1374	-0.71	-0.71	-0.72	0.72
EDI positive	*n* = 325	-0.08	-0.10	-0.15	0.18
QLF positive	*n* = 338	-0.11	-0.15	-0.26	0.30

Absolute values of Spearman correlation were found to be above 0.7 when all observations were considered. When restricting the computation to those observations in which EDI or QLF had detected lesions, the absolute values of Spearman correlation were at most 0.3.

Comparing the QLF values of these lesions, fluorescence loss (ΔF) was, on average, 0.4% lower for lesions undetectable by visual assessment. Lesions identified exclusively by QLF exhibited an average ΔQ value of -185%/px^2^ and an average size of 27.4 px^2^. By contrast, lesions detectable by both QLF and visual assessment exhibited a greater fluorescence loss (ΔQ of -514%/px^2^) and a larger lesion area (72.9 px^2^) (Table 5).

**Table 5:** Mean and median QLF values of lesions detected by QLF alone versus QLF combined with EDI

Measure		Diagnostic procedure	
		EDI + QLF (*n* = 259)	QLF (*n* = 79)
**ΔF**	Mean (SD)	-6.8 (1.3)	-6.4(2.2)
	Median (IQR)	-6.5 (-7.3, -5.9)	-6.2 (-6.8, -5.9)
**ΔFmax**	Mean (SD)	-10.0 (4.1)	-9.2, 4.8)
	Median (IQR)	-9.0 (-11.0, -7.0)	-8.0 (-10.0, -7.0)
**ΔQ**	Mean (SD)	-514 (1008)	-185, 174.0)
	Median (IQR)	-247 (-499, -84.0)	-124 (-252, -56.0)
**WS area**	Mean (SD)	72.9 (131.1)	27.4, 25.2)
	Median (IQR)	39.0 (14.0, 78.0)	19.0 (9.0, 36.0)

Cohen’s kappa coefficient was employed to assess the concordance between QLF and the EDI, following the methodology described by Banks. In this study, the kappa coefficient was 0.84 (95% CI = [0.79; 0.90]) at T0, 0.63 (95% CI = [0.54; 0.72]) at T1, and 0.65 (95% CI = [0.57; 0.73])at T2. When aggregating the data across all three time points, the overall kappa value was 0.71 (95% CI = [0.67; 076]), with an overall concordance rate of 89.4%.

**Fig. 4:**
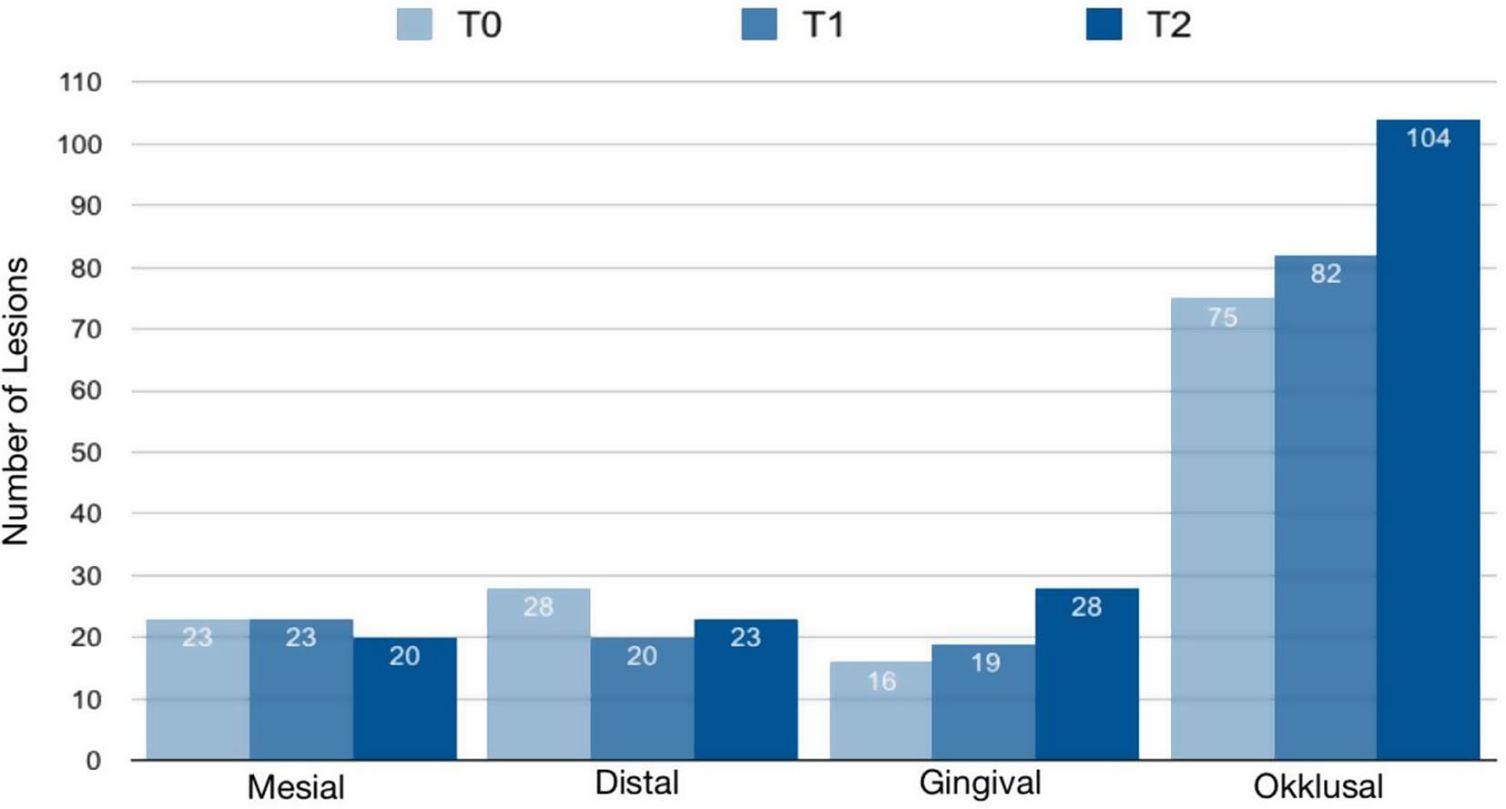
Prevalence of initial caries lesions according to the EDI (Banks). The bar chart illustrates the number of lesions on the buccal tooth surface at various time points, categorized by location

The EDI method divides each buccal tooth surface into four regions: mesial, distal, gingival, and occlusal location. Visual examination using the EDI revealed the greatest increase in lesions in the gingival and occlusal areas (Fig. 4). A total of 2076 partial surfaces were evaluated at T0, 1728 at T1 and 1692 at T2.

Prior to MB therapy, 93.2% (*n* = 1934) of surfaces exhibited no visible demineralization (grade 0). This figure decreased slightly to 91.7% (*n* = 1584) at T1 and further to 90.2% (*n* = 1527) at T2. Initially, 5.6% of surfaces (*n* = 117) presented with grade 1 lesions, which increased to 7.8% (*n* = 134) at T1, and 8.7% (*n* = 147) at T2, indicating demineralization affecting less than 50% of the surface. In summary, across all teeth consistently evaluated over all three time points, the modified EDI sum was calculated to be 0.32 (± 0.76) before MB therapy. This value rose to 0.36 (± 0.8) after six months, and further to 0.48 (± 0.88) one year following the initiation of therapy.

## Discussion

The diagnosis of initial caries lesions in clinical practice predominantly relies on visual-tactile examination. Beyond early lesion detection, the clinical applicability, reproducibility and diagnostic of any method are of utmost importance. In the present clinical study, the outcomes of visual-tactile examination were compared with those of QLF measurement. Both diagnostic techniques independently confirmed an increase in the prevalence and incidence of initial caries lesions within one year following the initiation of orthodontic treatment (Figs. 2 and 3). Notably, after the insertion of the MB appliances, the population of Streptococcus mutans increases significantly within three months [25], as the orthodontic apparatus facilitates bacterial adherence and colonization [26]. Accordingly, the study’s observation period was designed to align with the time frame in which caries-promoting factors - elevated during the active phase of therapy [10]– contribute to the formation of initial lesions.

The prevalence of initial caries lesions during orthodontic treatment has been extensively explored, with reported results ranging widely between 2 − 96% [1, 9, 11–13, 27]. The variability is largely attributable to substantial differences in study designs and analytical methodologies [28, 29]. Key variations include the duration of treatment, the age of participants, and the diagnostic approach employed.

In some investigations, intraoral photographs were used to apply the initial caries lesion index [14, 27], while in others, the index was directly determined through visual inspection of the subjects [9, 11, 30]. Additionally, factors such as ethnicity, participant selection and variations in bonding materials and oral hygiene protocols are known to influence initial caries lesion development [28, 31]. These methodological discrepancies may contribute to the divergence in study outcomes. Nonetheless, the studies consistently indicate that initial caries lesions increase during orthodontic treatment.

The distribution of lesions across individual teeth revealed the highest prevalence in the anterior maxillary region, with the central incisors being the most frequently affected by initial caries lesions (Figs. 2 and 3). This may be attributed to the early eruption of incisors, resulting in prolonged exposure to cariogenic factors within the oral cavity [32]. Conversely, the second premolars in both the maxillary and mandibular regions exhibited the lowest prevalence. In addition to their anatomical proximity to the excretory duct of the parotid gland – thus benefiting from the buffering effect of saliva, a key component in caries prevention - the reduced assessability of the teeth via QLF further contributed to the lower prevalence observed. As such, the selection of teeth for imaging using biluminator technology warrants careful consideration. The anterior teeth are more consistently assessable compared to premolars, which were frequently recorded as “not assessable” in this study. Issues such as poor image quality, gingival hyperplasia or incomplete eruption often led to the exclusion of certain tooth surfaces. When comparing diagnostic methods, no lesions were detected by either QLF or EDI on 970 of the 1374 tooth surfaces examined (70.6%). Both methods identified lesions on 259 surfaces (18.9%) (Fig. 4). The overall concordance between QLF and EDI for detecting initial caries lesions and fluorescence loss was 89.1% (*n* = 1229), with a Cohen’s kappa coefficient of 0.71.

While both methods have strong agreement in determining the presence or absence of lesions, the correlation between EDI score and QLF measurements is limited once presence of a lesion has been established by at least one method.

One advantage of the QLF method is its ability to visually present the dark areas to patients, thereby encouraging improved oral hygiene practices [22]. This visible feedback may enhance patient motivation and cooperation, as it allows them to monitor the progression of the lesions in real time [33]. In our study, QLF identified lesions on 79 tooth surfaces (5.8%) that were not detected by EDI. Heinrich-Weltzien et al. [34] posited that QLF’s heightened sensitivity enables earlier detection of initial caries lesions on smooth surfaces. Consistent with this, our findings revealed QLF values closer to zero for these lesions compared to those visible to the naked eye, aligning with a comparative study that reported QLF values of ΔF -9.6% for lesions detected by both EDI and QLF, and ΔF -7.1% for QLF-only detections [34].

However, the clinical significance of such findings must be critically evaluated, as they cannot be directly compared to studies correlating QLF data with the ICDAS and histological scores [35]. The primary aim of this study was to establish a practical framework for validating QLF scores [36]. For instance, an ICDAS-II score of 0 (indicating no visible caries after air drying) aligns with a QLF score of 0 (ΔF = -0.5 to -12), both of which denote healthy tooth structure. Similarly, an ICDAS-II score of 1 (representing initial visual changes in the enamel surface, visible only after drying, typically as opacities or slight discolorations) corresponds to a QLF score of 1 (ΔF = -12.5 to -18), as identified by Alammari et al. [35]. Furthermore, these QLF values substantiated by histological analyses, showing that at QLF values between − 10.5 and − 15, early enamel changes become discernible, while more pronounced alterations are evident at values exceeding − 15.5 [36].

Proper drying of the tooth surface is crucial for both QLF and visual examinations. QLF image acquisition is notably more time-consuming, requiring both photographic capture and subsequent analysis. Complete removal of saliva is essential for accurate QLF assessment, although this process can be challenging and time-intensive in some cases due to the need for coordination between the tongue, cheek retractors, and precise tooth positioning. Additionally, prolonged dehydration of enamel can skew QLF results by artificially increasing fluorescence loss [37].

Heinrich-Weltzien et al. [34] reported that 4.9% of lesions identified via visual examination were undetectable by the QLF method, citing confounding factors such as gingival hyperplasia, plaque accumulation, and suboptimal image quality. In our study, these variables were mitigated through professional dental cleaning and the exclusion of tooth surfaces that could not be adequately assessed. When a suspected lesion appeared as a darker area but was partially obscured by an orthodontic arch wire, accurate measurement was impeded. However, in-situ diagnosis using EDI was feasible by adjusting the viewing angle using mirrors, highlighting a limitation of the QLF technique. As a result, 4.8% (*n* = 66) of surfaces showed lesion only by visual inspection (EDI) but lacked corresponding fluorescence loss due to obstruction by the arch wire.

The acquisition of QLF images requires trained personnel to ensure consistent angulation and accurate progression analysis. For QLF to be reliably integrated into routine clinical practice, it is imperative to minimize the influence of extraneous factors that can compromise diagnostic accuracy.

It is important to recognize that the time-intensive and complex QLF examination of each tooth surface for clinically invisible lesions presents practical limitations. While early detection and quantification of lesions are essential for optimizing interceptive measures, QLF analysis of inactive lesions with intact surface integrity appears impractical. Conversely, active lesions, which are more commonly located near the gingival margin, are particularly challenging to capture and analyze using QLF [34]. Additionally, QLF exhibits a reduced effectiveness in detecting lesions in areas that are difficult to access or obscured [38] and relies on the presence of sound enamel surrounding the respective lesion as a reference for fluorescence values [39].

## Conclusion

The findings of this study underscore the potential of the QLF method as a sensitive diagnostic tool while highlighting its limitation in accessing challenging tooth surfaces, particularly in the presence of orthodontic appliances. Both the QLF and EDI methods demonstrated comparative effectiveness in the detection and analysis of initial caries lesions. Given the reliability of both approaches, future considerations should weigh the cost-effectiveness and time-efficiency of each method to determine the most appropriate option for individual cases.

### Clinical relevance

The comparable performance of QLF and EDI underscores their efficacy and reliability as diagnostic tools. However, it is important to acknowledge that while QLF is highly sensitive, its application may be difficult in certain clinical situations.

## Data Availability

No datasets were generated or analysed during the current study.
